# Kaempferol Ameliorates Oxygen-Glucose Deprivation/Reoxygenation-Induced Neuronal Ferroptosis by Activating Nrf2/SLC7A11/GPX4 Axis

**DOI:** 10.3390/biom11070923

**Published:** 2021-06-22

**Authors:** Yuan Yuan, Yanyu Zhai, Jingjiong Chen, Xiaofeng Xu, Hongmei Wang

**Affiliations:** Department of Neurology, Shanghai Jiao Tong University Affiliated Sixth People’s Hospital, Shanghai 200233, China; 767761297@sjtu.edu.cn (Y.Y.); 20141490@stu.nun.edu.cn (Y.Z.); jjiong76@yeah.net (J.C.); xuxiaofeng987620@126.com (X.X.)

**Keywords:** ischemic stroke, kaempferol, Nrf2, GPX4, lipid peroxidation, ferroptosis

## Abstract

Kaempferol has been shown to protect cells against cerebral ischemia/reperfusion injury through inhibition of apoptosis. In the present study, we sought to investigate whether ferroptosis is involved in the oxygen-glucose deprivation/reperfusion (OGD/R)-induced neuronal injury and the effects of kaempferol on ferroptosis in OGD/R-treated neurons. Western blot, immunofluorescence, and transmission electron microscopy were used to analyze ferroptosis, whereas cell death was detected using lactate dehydrogenase (LDH) release. We found that OGD/R attenuated SLC7A11 and glutathione peroxidase 4 (GPX4) levels as well as decreased endogenous antioxidants including nicotinamide adenine dinucleotide phosphate (NADPH), glutathione (GSH), and superoxide dismutase (SOD) in neurons. Notably, OGD/R enhanced the accumulation of lipid peroxidation, leading to the induction of ferroptosis in neurons. However, kaempferol activated nuclear factor-E2-related factor 2 (Nrf2)/SLC7A11/GPX4 signaling, augmented antioxidant capacity, and suppressed the accumulation of lipid peroxidation in OGD/R-treated neurons. Furthermore, kaempferol significantly reversed OGD/R-induced ferroptosis. Nevertheless, inhibition of Nrf2 by ML385 blocked the protective effects of kaempferol on antioxidant capacity, lipid peroxidation, and ferroptosis in OGD/R-treated neurons. These results suggest that ferroptosis may be a significant cause of cell death associated with OGD/R. Kaempferol provides protection from OGD/R-induced ferroptosis partly by activating Nrf2/SLC7A11/GPX4 signaling pathway.

## 1. Introduction

Ischemic stroke is a major cause of mortality and serious disability worldwide, accounting for about 80% of stroke. Ischemic stroke occurs when a blood vessel is blocked, leading to acute disrupted cerebral blood flow and oxygen delivery to the brain. However, the underlying mechanisms of brain injury in ischemic stroke remain poorly understood. More recently, ferroptosis is described as an iron-dependent form of cell death mediated by excess accumulation of lipid peroxides [1,2]. Glutathione peroxidase 4 (GPX4) is a key regulator of ferroptosis and plays a crucial role in converting lipid hydroperoxides to non-toxic lipid [3,4]. Besides, glutathione (GSH) acts as an essential cofactor for GPX4. Importantly, inactivation of GPX4 or depletion of GSH causes the accumulation of lipid hydroperoxides, eventually leading to the induction of ferroptosis [3,5,6]. Moreover, neuron-specific GPX4 depletion in the forebrain elevates lipid peroxidation, increases ferroptosis, and triggers neurodegeneration [7]. Additionally, ferroptosis can be induced by disrupting the function of cystine/glutamate antiporter system x_c_^-^ [8]. Inhibition of SLC7A11, the light chain of system x_c_^-^, attenuates GSH levels and GPX4 activity, resulting in the accumulation of lethal lipid peroxides and the induction of ferroptosis [2,9]. Nuclear factor-E2-related factor 2 (Nrf2) is a key transcription factor that controls cellular redox homeostasis and inflammation. Activation of Nrf2 signaling also protects cells against ferroptosis in many chronic diseases [10,11]. In addition, Nrf2 acts as a transcription factor increasing the expression of SLC7A11 and GPX4 [8,10,11]. Accumulating evidence reveals that ferroptosis is involved in various pathological conditions including neurodegenerative disease, traumatic brain injury, and stroke [12,13]. 

Moreover, kaempferol (KF) is a major bioflavonoid found in many fruits, vegetables, and medicinal plants. Interestingly, kaempferol has neuroprotective, antioxidant, and anti-cancer properties against many diseases associated with lipid oxidation, including stroke, Alzheimer’s disease, and cancer [14,15,16]. 

In the present study, we show that ferroptosis is involved in oxygen-glucose deprivation/reperfusion (OGD/R)-induced cell death and kaempferol protects neurons from OGD/R-induced cell injury partly by activating Nrf2/SLC7A11/GPX4 signaling pathway and eventually inhibiting the onset of ferroptosis. Hence, ferroptosis is a significant cause of cell death associated with ischemia/reperfusion (I/R) injury and kaempferol has considerable potential for the treatment of ischemia stroke.

## 2. Materials and Methods

### 2.1. Chemicals

Kaempferol (KF) was purchased from Sigma (St. Louis, MO, USA). ML385 (Nrf2 inhibitor) was provided by MedChemExpress (Monmouth Junction, NJ, USA). 3-(4,5-dimethylthiazol-2-yl)-2,5-diph-enyltetrazoliumbromide (MTT) and all materials related to cell cultures were obtained from Thermo Fisher Scientific (Waltham, MA, USA).

### 2.2. Primary Cortical Neuron Cultures

Primary mouse cortical neurons were prepared from E16 mouse embryos (C57BL/6j mice) as previously described [17]. In brief, dissected cerebral cortices were enzymatically digested. Dissociated neurons were grown in neurobasal medium supplemented with 2% B27 and 2 mM glutamax. Cultures were maintained at 37 °C in a humidified incubator with 5% CO_2_. The experiments were performed on day in vitro (DIV) 10–12. 

### 2.3. Oxygen Glucose-Deprivation/Reperfusion (OGD/R) and Drug Treatment

Primary neurons were cultured with glucose-free DMEM and placed in a hypoxic incubator filled with mixed gas containing 1% O_2_, 5% CO_2_, and 94% N_2_ at 37 °C for 90 min to induce OGD injury. Following OGD, the medium was replaced by fresh neurobasal medium, and neurons were incubated in a 37 °C/5% CO_2_ incubator for a 24 h reperfusion period. Control neurons were grown in glucose-containing media in a normoxic incubator for the same time period. Neurons were divided into four groups: control, OGD/R, OGD/R + KF, and OGD/R + KF + ML385 group. Neurons were untreated or stimulated with OGD/R, or pretreated with KF (10 µM) or KF (10 µM) plus ML385 (1 µM) for 1 h and followed by exposure to OGD/R. Kaempferol was dissolved in DMSO. All treatments were performed using the same concentration of DMSO (0.1%). Besides, the same amount of DMSO (0.1%) was added to control neurons.

### 2.4. Measurement of Intracellular Reactive Oxygen Species (ROS) Levels

Intracellular ROS levels were detected using CM-H2DCFDA (Molecular Probes, Eugene, OR, USA). Neurons were stained with 10 µM CM-H2DCFDA in the dark for 30 min at 37 °C and the fluorescence intensity was measured using a microplate reader. Each experiment was measured in triplicate.

### 2.5. Lipid ROS Assay

Lipid ROS levels were measured using C11-BODIPY 581/591 (Thermo Fisher Scientific, Waltham, MA, USA). Neurons were incubated with 2.5 μM C11-BODIPY 581/591 in the dark for 30 min at 37 °C. The fluorescence intensity was then assessed using a microplate reader with an excitation wavelength of 485 nm and an emission wavelength of 520 nm [18]. Each experiment was measured in triplicate.

### 2.6. MTT Assay

Cell viability was tested using MTT assay as previously described [17]. Briefly, the medium was removed from treated neurons and subsequently replaced with MTT reagent (0.5 mg/mL). After a 4 h incubation at 37 °C, the MTT solution was removed and replaced with 100 μL dimethyl sulfoxide (DMSO) to dissolve the formazan. The absorbance of the 96-well plate was recorded at 570 nm using a microplate reader. Each experiment was measured in triplicate.

### 2.7. CCK-8 Assay

Cellular proliferation was also investigated with the CCK-8 assay (Dojindo, Kumamoto, Japan) according to the manufacturer’s instructions. Each experiment was measured in triplicate.

### 2.8. LDH Assay

Lactate dehydrogenase (LDH) release from cells was determined using the lactate dehydrogenase detection kit (Sigma, St. Louis, MO, USA) according to the manufacturer’s instructions. Each experiment was measured in triplicate.

### 2.9. Measurement of GPX4 Activity 

Glutathione peroxidase 4 (GPX4) activity was performed as described previously [19]. The GPX4 activity was tested using a buffer with 0.1 M KH_2_ PO_4_/K_2_ HPO_4_, 5 mM EDTA, 0.1% (*v*/*v*) Triton X-100, 5 mM GSH, 160 mM NADPH/H^+^, and 180 IU/mL glutathione reductase. 5 µL of 30 mM cumene hydroperoxide was added to initiate the reaction. NADPH oxidation was then detected at 340 nm for 10 min. Each experiment was measured in triplicate.

### 2.10. GSH/GSSG Ratio and NADPH/NADP^+^ Ratio Measurements

The reduced glutathione (GSH)/oxidized glutathione (GSSG) ratio and the NADPH/NADP^+^ ratio were analyzed using GSH/GSSG ratio detection assay kit (Abcam, Cambridge, UK) and NADP/NADPH assay kit (Sigma, St. Louis, MO, USA), following the manufacturer’s instructions. Each experiment was measured in triplicate.

### 2.11. Measurements of Lipid Peroxidation and SOD Activity

Levels of malondialdehyde (MDA) and 4-hydroxynonenal (4-HNE) were assessed using the MDA assay kit (Jiancheng Bioengineering Institute, Nanjing, China) and the 4-HNE assay kit (Abcam, Cambridge, UK) according to the manufacturer’s protocols. Superoxide dismutase (SOD) activity was determined using the SOD assay kit (Jiancheng Bioengineering Institute, Nanjing, China) following the manufacturer’s protocol.

### 2.12. Measurement of Intracellular Iron 

Intracellular iron concentrations were determined using the iron assay kit (Abcam, Cambridge, UK) following the manufacturer’s instructions.

### 2.13. Immunofluorescence

Cells were fixed with 4% paraformaldehyde for 15 min and permeabilized with 0.2% Triton X100. Cells were blocked with 10% goat serum for 30 min and then incubated with (SLC7A11 or GPX4) primary antibodies at 4 °C overnight. After washing in PBS, cells were incubated with Alexa Fluor 594 secondary antibody (Molecular Probes, Eugene, OR, USA) at room temperature for 1 h. Nuclei were stained with DAPI (Sigma, St. Louis, MO, USA) for 10 min. Pictures were subsequently taken on a confocal microscope or a fluorescence microscope.

### 2.14. Western Blots Analysis

After treatment, cells were collected and lysed in 1× RIPA lysis buffer containing protease inhibitor cocktail. Nuclear and cytoplasmic fractions were isolated using a nuclear and cytoplasmic protein extraction kit (Beyotime Biotechnology, Shanghai, China) according to the manufacturer’s instructions. Samples (20 µg per lane) were separated in SDS-PAGE gels and transferred to a PVDF membrane. The membrane was blocked with TBST buffer containing 5% nonfat milk for 1 h at room temperature and incubated with primary antibodies against SLC7A11 (Abcam, Cambridge, UK), GPX4 (Abcam, Cambridge, UK), Nrf2 (Abcam, Cambridge, UK), 4-HNE (Abcam, Cambridge, UK), β-actin (Abcam, Cambridge, UK) and Histone-3 (Cell Signaling Technology, Beverly, MA, USA) overnight at 4 °C. Secondary antibodies (Santa Cruz, CA, USA) were diluted in 5% nonfat milk in TBST and then added for 1 h at room temperature. Bands were developed using ECL detection reagent.

### 2.15. Transmission Electron Microscopy (TEM)

Neurons were collected and immediately fixed in a solution containing 2% glutaraldehyde for 2 h. The samples were then post-fixed with 1% osmium tetroxide. After dehydration through a graded ethanol series, the cells were embedded in Epon Resin 618. Ultrathin sections were subsequently stained with uranyl acetate and lead citrate. Images were collected using a Philips CM120 transmission electron microscope.

### 2.16. Statistical Analysis 

Statistical analyses were performed using Prism 5.0 GraphPad Software. All experimental measurements were repeated at least three times, and the data were presented as the means ± SEM. The values were analyzed using one-way ANOVA with Bonferroni’s post hoc test for multiple comparisons. A value of *p* < 0.05 was considered statistically significant.

## 3. Results

### 3.1. Kaempferol Activates SLC7A11, GPX4, and Nrf2 in OGD/R-Treated Neurons

**Figure 1:**
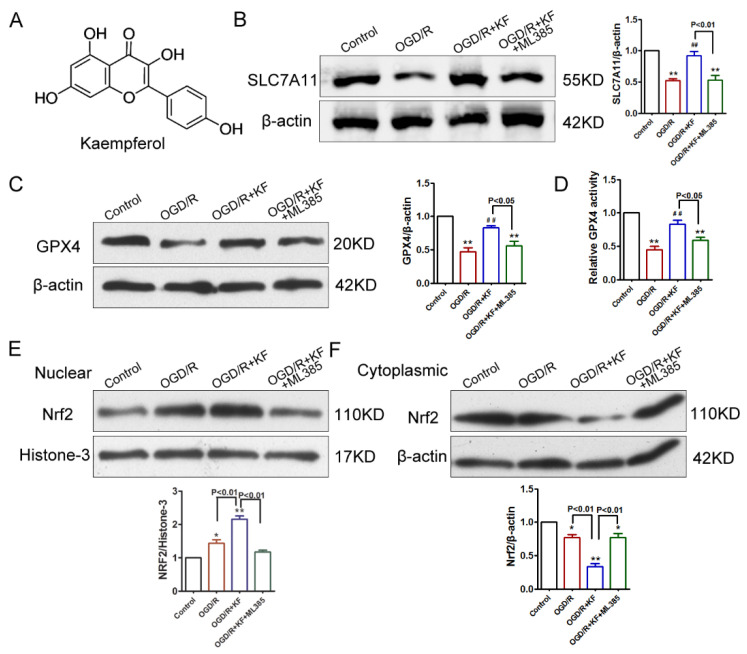
Effects of kaempferol on GPX4, SLC7A11, and Nrf2 levels in OGD/R-treated neurons. (**A**) Chemical structure of kaempferol (KF). (**B**) Neurons were untreated or stimulated with OGD/R, or pretreated with KF (10 µM) or KF (10 µM) plus ML385 (1µM) for 1 h prior to treatment with OGD/R. Then SLC7A11 protein levels were detected by Western blot. β-actin was used as a loading control. Western blot is representative of three independent experiments. (**C**) GPX4 protein levels were detected by Western blot. (**D**) GPX4 activity was measured. (E) Nuclear Nrf2 protein levels were detected by Western blot. Histone-3 was used as a loading control for nuclear fractions. (**F**) Cytoplasmic Nrf2 protein levels were detected by Western blot. Data were averaged from three independent experiments.* *p* < 0.05 versus control; ** *p* < 0.01 versus control; ^##^ *p* < 0.01 versus OGD/R.

**Figure 2:**
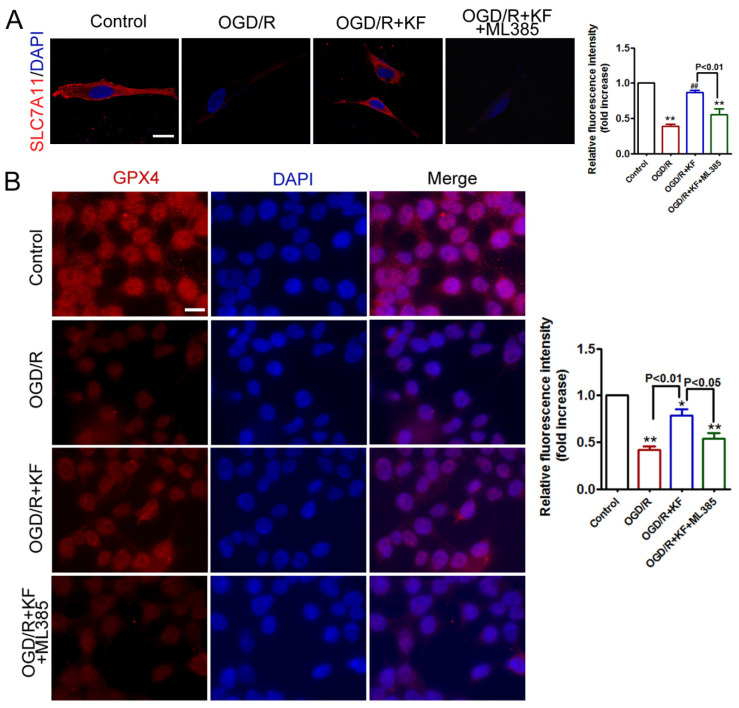
Effects of kaempferol on GPX4 and SLC7A11 expression in OGD/R-treated neurons. (**A**) Neurons were treated as indicated in (Figure 1A). Neurons were then immunostained using antibody specific for SLC7A11 (red). Nucleus is stained with DAPI (blue). Representative confocal images of the indicated neurons from three independent experiments. Scale bar, 15 µm. (**B**) Neurons were treated as indicated in (Figure 1A). Neurons were then immunostained using antibody specific for GPX4 (red). Nucleus is stained with DAPI (blue). Images were acquired using a fluorescence microscope. Representative immunostaining images of the indicated neurons from three independent experiments. Scale bar, 15 µm. * *p* < 0.05 versus control; ** *p* < 0.01 versus control; ^##^ *p* < 0.01 versus OGD/R.

Kaempferol (Figure 1A) has been demonstrated to protect cells against ischemia stroke by inhibiting apoptosis, neuroinflammation, and blood-brain barrier (BBB) disruption [20,21]. However, it remains unclear whether kaempferol suppresses OGD/R-induced ferroptosis. GPX4 is very important for regulating ferroptosis and SLC7A11 acts as an upstream mediator of GPX4 [22]. In addition, Nrf2 can inhibit ferroptosis via regulation of SLC7A11 [10,23]. To determine whether OGD/R-induced injury was associated with ferroptosis in neuron, we investigated protein levels of SLC7A11, GPX4, and Nrf2 by Western blots. As shown in Figure 1B,C, OGD/R obviously decreased SLC7A11 and GPX4 protein levels, which were rescued by kaempferol (10 μM). The concentration of kaempferol used in the present study was determined based upon published studies [24,25]. Consistent with the result of GPX4 expression, reduced GPX4 activity was detected in neurons exposed to OGD/R (Figure 1D). Besides, OGD/R insult slightly increased nuclear Nrf2 level and attenuated cytoplasmic Nrf2 level in neurons (Figure 1E,F). However, kaempferol not only strongly enhanced protein levels of SLC7A11, GPX4, and nuclear Nrf2 but also dramatically suppressed cytoplasmic Nrf2 level in OGD/R-treated neurons. Importantly, kaempferol strikingly elevated GPX4 activity in neurons subjected to OGD/R insult. Conversely, Nrf2 inhibitor ML385 (1 μM) significantly abrogated the effects of kaempferol on protein levels of SLC7A11, GPX4, and Nrf2 as well as GPX4 activity in OGD/R-treated neurons (Figure 1B–F). SLC7A11 and GPX4 expression was further assessed by immunofluorescence. In line with protein levels of SLC7A11 and GPX4, OGD/R significantly attenuated expression of SLC7A11 and GPX4, which was rescued by kaempferol (Figure 2A,B). Compared with OGD/R group, expression of SLC7A11 and GPX4 was not changed in OGD/R + KF + ML385 group. Additionally, ML385 inhibited beneficial effects of kaempferol on the expression of SLC7A1 and GPX4 (Figure 2A,B) in OGD/R-treated neurons. 

### 3.2. Kaempferol Exhibits Antioxidant Effects and Ameliorates OGD/R-Induced Fe^2+^ Accumulation in Neurons

**Figure 3:**
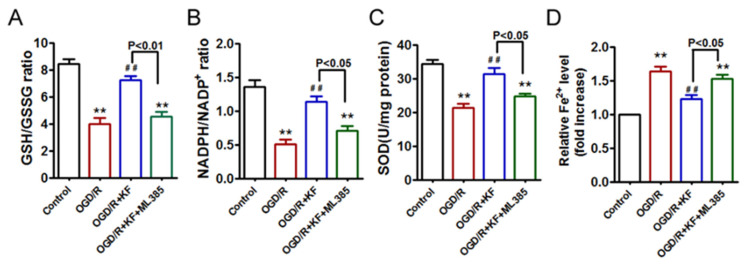
Effects of kaempferol on endogenous antioxidants and intracellular iron content in OGD/R-treated neurons. Neurons were treated as indicated in (Figure 1A). GSH/GSSG ratio (**A**), NADPH/NADP+ ratio (**B**), SOD activity (**C**), and intracellular iron content (**D**) were tested by ELISA. Three independent experiments were performed with similar results. ** *p* < 0.01 versus control; ^##^ *p* < 0.01 versus OGD/R.

GSH, NADPH, and SOD are antioxidant markers. We then detected the effects of kaempferol on ratios of GSH/GSSG and NADPH/NADP^+^ as well as SOD activity. OGD/R insult obviously suppressed GSH/GSSG and NADPH/NADP^+^ ratios, which were reversed by kaempferol (Figure 3A,B). However, there were no significant differences in the ratios of GSH/GSSG and NADPH/NADP^+^ between OGD/R group and OGD/R + KF + ML385 group. Interestingly, OGD/R-treated neurons displayed decreased SOD activity in comparison to control neurons, whereas kaempferol significantly improved SOD activity inhibited by OGD/R insult (Figure 3C). Compared with OGD/R + KF group, SOD activity was reduced in OGD/R + KF + ML385 group. These results suggested that ML385 reverses the favorable effects of kaempferol on oxidative stress in OGD/R-treated neurons. Given that ferroptosis is an iron-dependent form of cell death, we also detected intracellular iron levels in neurons. As expected, OGD/R significantly increased Fe^2+^ levels (Figure 3D), which were abolished by kaempferol. Nevertheless, ML385 plus kaempferol pretreatment failed to attenuate OGD/R-induced Fe^2+^ accumulation.

### 3.3. Kaempferol Inhibits OGD/R-Induced Lipid Peroxidation in Neurons

**Figure 4:**
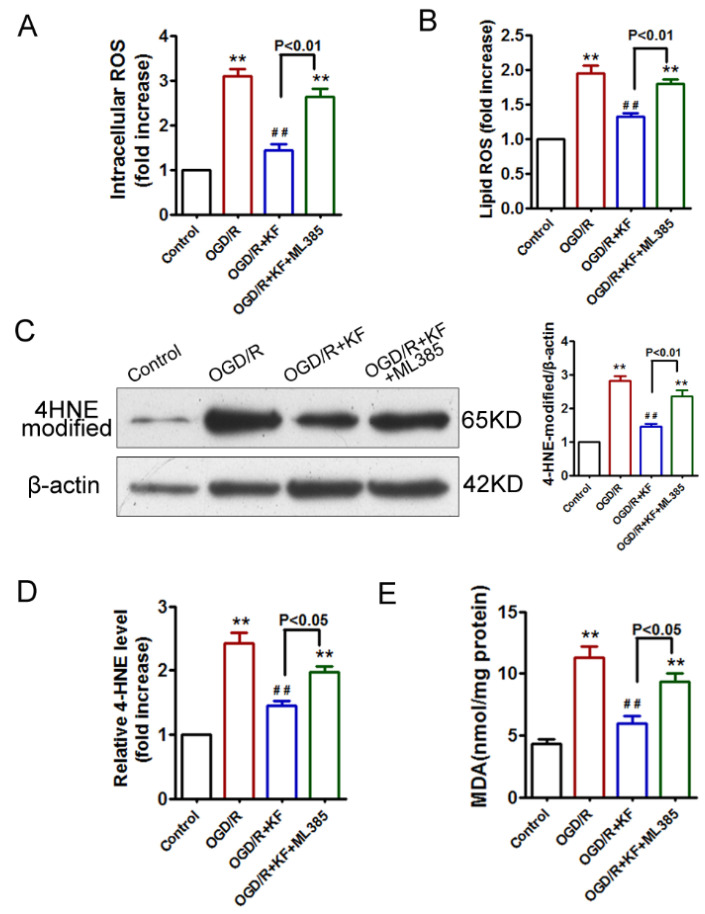
Effects of kaempferol on ROS, 4-HNE, and MDA levels in OGD/R-treated neurons. Neurons were treated as indicated in (Figure 1A). (**A**) Intracellular ROS production was measured with DCFH-DA. (**B**) Lipid ROS level was assessed by BODIPY-C11 staining. (**C**) 4-HNE-modified protein levels were detected by Western blot. (**D**) Levels of 4-HNE were tested by ELISA. Three independent experiments were performed with similar results. (**E**) Levels of MDA were investigated by ELISA. ** *p* < 0.01 versus control; ^##^ *p* < 0.01 versus OGD/R.

It has been shown that lipid ROS accumulation is a hallmark of ferroptosis [26,27]. Therefore, we investigated the effects of kaempferol on intracellular and lipid ROS accumulation in OGD/R-treated neurons. OGD/R induced the accumulation of intracellular and lipid ROS, whereas kaempferol remarkably inhibited intracellular and lipid ROS accumulation triggered by OGD/R insult (Figure 4A,B). However, ML385 abrogated the beneficial effects of kaempferol on intracellular and lipid ROS accumulation. 4-HNE is the end product of lipid peroxidation that can readily modify proteins [28,29,30]. 4-HNE has been identified as a key second messenger involved in the regulation of cell proliferation, ferroptosis, and apoptosis. Although less is known about protein adducts with 4-HNE, increased levels of 4-HNE protein adducts are associated with enhanced oxidative damage [28,29,30]. As expected, 4-HNE-modified protein expression (4-HNE protein adducts) was increased in response to OGD/R insult, which was significantly abolished by kaempferol pretreatment (Figure 4C). Conversely, there was no significant difference in the 4-HNE-modified protein expression between OGD/R group and OGD/R + KF + ML385 group. As shown in Figure 4D, kaempferol obviously suppressed OGD-triggered 4-HNE activity, whereas no significant difference in 4-HNE activity was detected between OGD/R group and OGD/R + KF + ML385 group. Notably, ML385 enhanced 4-HNE-modified protein expression and activity inhibited by kaempferol. MDA, a marker of lipid peroxidation, was also measured [31]. Similarly, kaempferol pretreatment significantly inhibited MDA levels augmented by OGD/R insult, whereas kaempferol plus ML385 failed to suppress MDA levels in OGD/R-treated neurons (Figure 4E). These results confirmed that activation of Nrf2 by kaempferol attenuated OGD/R-induced lipid peroxidation in neurons.

### 3.4. Kaempferol Attenuates OGD/R-Induced Ferroptosis in Neurons

**Figure 5:**
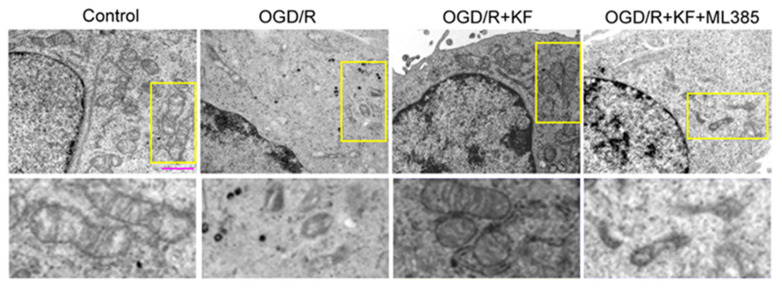
Effect of kaempferol mitochondrial morphology in OGD/R-treated neurons. Neurons were treated as indicated in (Figure 1A). Mitochondrial morphology associated with ferroptosis was determined by transmission electron microscopy. Bottom panels display the magnified images of regions indicated by yellow rectangles in the top panels. Scale bar, 1 µm.

Next, we observed the morphological changes of mitochondria under transmission electron microscopy. OGD/R-treated neurons displayed fragmented mitochondria with decreased cristae and increased mitochondrial membrane density, which confirmed that OGD/R induces ferroptosis [32,33] (Figure 5). However, kaempferol obviously inhibited morphological changes of mitochondria in response to OGD/R. Mitochondria in neurons treated with OGD/R plus kaempferol appeared intact mitochondrial cristae. Conversely, ML385 reversed the favorable effects of kaempferol on morphological changes of mitochondria in OGD/R-treated neurons (Figure 5). 

### 3.5. Kaempferol Protects Neurons against OGD/R-Induced Cell Death

**Figure 6:**
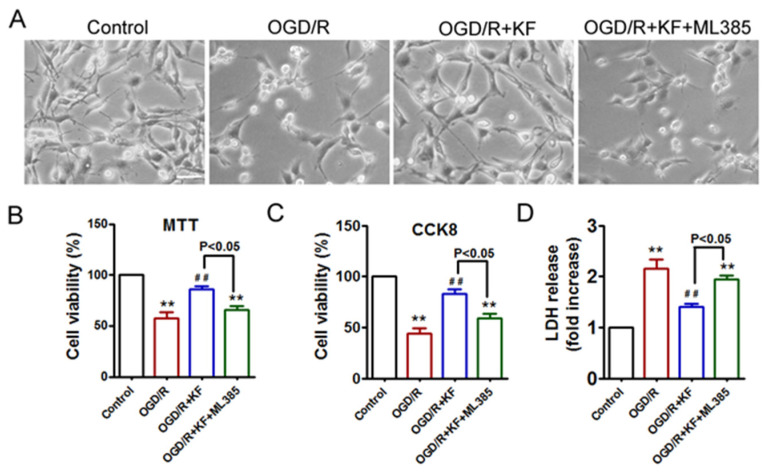
Effects of kaempferol on cell viability and cell death in OGD/R-treated neurons. Neurons were treated as indicated in (Figure 1A). (**A**) Phase contrast imaging of live neurons. Scale bar, 50 µm. (**B**–**D**) Cell viability and cell death were measured by MTT assay (**B**), CCK-8 (**C**), and LDH (**D**), respectively. Three independent experiments were performed with similar results. ** *p* < 0.01 versus control; ^##^ *p* < 0.01 versus OGD/R.

**Figure 7:**
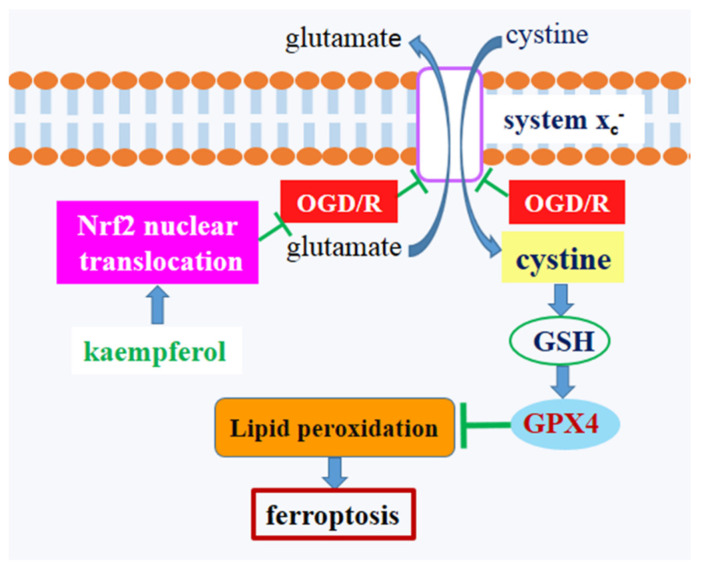
Schematic diagram showing the protective mechanism of kaempferol against OGD/R-induced ferroptosis in neurons.

In order to clarify the protective effects of kaempferol on OGD/R-induced neuronal injury, we evaluated cell morphology, cell viability, and cell death in neurons exposed to OGD/R. Kaempferol rescued aberrant cell morphology of OGD/R-treated neurons, whereas kaempferol plus ML385 did not reverse abnormal cell morphology in neurons subjected to OGD/R insult (Figure 6A). Furthermore, results of MTT, CCK-8, and LDH showed that OGD/R attenuated cell viability and increased cell death, which were significantly inhibited by the addition of kaempferol (Figure 6B–D). On the contrary, ML385 plus kaempferol pretreatment failed to promote cell survival in OGD/R-treated neurons. Compared with OGD/R + KF group, decreased cell viability and increased cell death were detected in OGD/R + KF + ML385 group. These results confirmed that kaempferol exerts protective effects on OGD/R-induced neuronal injury, which is blocked by Nrf2 inhibitor ML385. Model illustrating the mechanism by which kaempferol suppresses OGD/R-induced ferroptosis in neurons (Figure 7).

## 4. Discussion

Kaempferol is widely distributed in many fruits and vegetables. It is thought to exert anti-inflammatory, antioxidant, and anti-cancer effects in in-vitro studies [15,34]. Moreover, it exhibits neuroprotective benefits in animal studies [20,21]. Recent studies demonstrate that kaempferol protects cells from apoptosis associated with ischemia stroke [16,20,21]. Indeed, ferroptosis is also involved in the pathogenesis of stroke. However, little is known about whether kaempferol inhibits OGD/R-induced ferroptosis. 

Ferroptosis is a regulated form of cell death characterized by iron-dependent accumulation of lipid peroxidation. GPX4, an antioxidant defense enzyme, is a key regulator of ferroptosis in multiple cell types. GXP4 ablation induces ferroptosis and triggers neurodegeneration in forebrain neurons [7]. Inhibition of GPX4 promotes ferroptosis in cancer cells [35,36]. GPX4 and its co-factor GSH are important for eliminating lipid peroxidation. It is well-established that GPX4 can reduce hydroperoxides at the expense of GSH [37]. In addition, the cystine/glutamate antiporter SLC7A11 is the major source of cysteine for GSH synthesis [9,26]. Besides, Nrf2 is a critical antioxidant transcription factor that regulates the expression of SLC7A11 and GPX4 [8,10,11]. Therefore, we observed the effects of kaempferol on protein levels of SLC7A11, GPX4, and Nrf2 in an in vitro stroke model of OGD/R. In vitro stroke model has many advantages in the ability to control experimental conditions and choose a specific region of the brain. As expected, OGD/R significantly decreased protein levels of SLC7A11 and GPX4, whereas it slightly increased nuclear Nrf2 level and attenuated cytoplasmic Nrf2 level in OGD/R-treated neurons. Increase nuclear translocation of Nrf2 after OGD/R insult may be an endogenous self-protective response. Importantly, protein levels of SLC7A11 and GPX4 were strikingly enhanced by kaempferol in OGD/R-treated neurons. Besides, kaempferol obviously increased nuclear Nrf2 level and suppressed cytoplasmic Nrf2 level in OGD/R-treated neurons, confirming that kaempferol triggers Nrf2 nuclear translocation. However, Nrf2 inhibitor ML385 abolished the protective effects of kaempferol on protein levels of SLC7A11, GPX4, and Nrf2 in OGD/R-treated neurons. These findings demonstrated that kaempferol activates Nrf2 and its subsequent downstream signaling cascades involving SLC7A11 and GPX4. Notably, decreased GPX4 activity was reversed by kaempferol in OGD/R-treated neurons. Nevertheless, ML385 abrogated the beneficial effect of kaempferol on GPX4 activity, supporting that Nrf2 acts as an upstream mediator of GPX4 in OGD/R-treated neurons. Besides, expression of SLC7A11 and GPX4 was also assessed by immunofluorescence. Kaempferol significantly rescued expression of SLC7A11 and GPX4 inhibited by OGD/R. Conversely, ML385 plus kaempferol failed to alter expression of SLC7A11 and GPX4 in neurons subjected to OGD/R. Taken together, these results supported that kaempferol activates Nrf2/SLC7A11/GPX4 signaling in OGD/R-treated neurons [16]. 

NADPH is critical for the conversion of oxidized glutathione (GSSG) to reduced glutathione (GSH) [38]. The reduced form of NADPH and GSH, as well as SOD are the most important endogenous antioxidants [38,39,40,41,42]. Exposure of OGD/R markedly decreased cellular NADPH/NADP^+^ and GSH/GSSG ratios, as well as SOD activity in neurons, indicating that OGD/R insult impairs antioxidant capacity. However, kaempferol not only elevated ratios of GSH/GSSG and NADPH/NADP^+^ but also enhanced SOD activity in OGD/R-treated neurons, confirming that kaempferol exhibits antioxidant effects. In contrast, ML385 reversed the antioxidant effects of kaempferol in neurons exposed to OGD/R, suggesting that activation of Nrf2 by kaempferol prevents OGD/R-induced oxidative stress. Moreover, OGD/R significantly increased Fe^2+^ level, which was abolished by kaempferol, whereas ML385 plus kaempferol did not reduce Fe^2+^ level in OGD/R-treated neurons, indicating that activation of Nrf2 by kaempferol might suppress OGD/R-induced ferroptosis.

Kaempferol has been shown to protect cells against death associated with cerebral ischemia/reperfusion injury [16,20,21]. Hence, we measured whether anti-ferroptosis is involved in the protective effects of kaempferol on OGD/R-induced cell death. The accumulation of lipid ROS is an important indicator of ferroptosis. We found that OGD/R significantly augmented intracellular and lipid ROS in neurons, confirming that OGD/R induces the occurrence of ferroptosis. Moreover, OGD/R-induced intracellular and lipid ROS accumulation was attenuated by kaempferol. However, suppression of Nrf2 by ML385 reversed the inhibitory effects of kaempferol on intracellular and lipid ROS accumulation. Besides, OGD/R enhanced 4-HNE and MDA activities in neurons, which were reduced by kaempferol. Conversely, ML385 plus kaempferol had no effect on 4-HNE and MDA activities in OGD/R-treated neurons. These results showed that Nrf2/SLC7A11/GPX4 signaling pathway is involved in the accumulation of lipid peroxidation and the induction of ferroptosis in neurons exposed to OGD/R insult. Nevertheless, activation of Nrf2/SLC7A11/GPX4 signaling by kaempferol attenuates ferroptosis in OGD/R-treated neurons. 

Additionally, transmission electron microscopy analysis showed that OGD/R promoted ferroptosis in neurons. Mitochondria in OGD/R-treated neurons appeared reduced cristae and increased mitochondrial membrane density, whereas kaempferol could significantly improve the abnormal morphology of mitochondria in OGD/R-treated neurons. Nevertheless, ML385 blocked the protective effects of kaempferol on OGD/R-induced ferroptosis. We subsequently investigated the effect of kaempferol on OGD/R-induced cell death. OGD/R-treated neurons displayed aberrant cell morphology, which was rescued by kaempferol. However, kaempferol plus ML385 did not alter abnormal cell morphology of OGD/R-treated neurons. We further found that OGD/R decreased cell viability and increased cell death in neurons. Conversely, kaempferol enhanced cell viability as well as ameliorated cell death in OGD/R-treated neurons, suggesting that kaempferol protects neurons from OGD/R-induced cell death. Nevertheless, OGD/R-induced cell death cannot be inhibited by ML385 plus kaempferol, indicating that pharmacological inhibition of Nrf2 abolishes the favorable effects of kaempferol. These results indicated that ferroptosis contributes to neuronal injury related to OGD/R.

## 5. Conclusions

In summary, ferroptosis may play a prominent role in cell death associated with ischemia/reperfusion injury. OGD/R inhibits SLC7A11 and GPX4, resulting in the induction of ferroptosis in neurons. Kaempferol provides protection from OGD/R-induced ferroptosis, at least in part, by activating Nrf2/SLC7A11/GPX4 signaling pathway. Therefore, pharmacological inhibition of ferroptosis may be an attractive therapeutic target for the treatment of ischemic stroke.

## Data Availability

Data is contained within the article.
